# Stratification of Sacroiliac Joint Pain Patients and the Efficacy of Ultrasound-Guided Injection Treatment: A Retrospective Study

**DOI:** 10.7759/cureus.86643

**Published:** 2025-06-24

**Authors:** Rita F Ferreira, André Dias, Ana Lídia Dias, Margarida Barbosa

**Affiliations:** 1 Anesthesiology, Faculdade de Medicina da Universidade do Porto, Porto, PRT; 2 Anesthesiology and Perioperative Medicine, Unidade Local de Saúde de São João, Porto, PRT; 3 CINTESIS-Center for Health Technology and Services Research, Faculdade de Medicina da Universidade do Porto, Porto, PRT; 4 Anesthesiology, Centro Hospitalar Universitário São João, Porto, PRT

**Keywords:** diagnosis, efficacy, injection, pain, sacroiliac joint, ultrasound

## Abstract

Introduction: Sacroiliac joint (SIJ) pain is a significant contributor to low back pain and affects patients´ quality of life. This study aims to (1) stratify patients with SIJ pain based on their clinical history and imaging findings, (2) evaluate the efficacy of ultrasound-guided injection techniques for pain management, and (3) identify predictors of pain relief.

Materials and methods: ​A retrospective observational study was conducted at the chronic pain unit (CPU) of a tertiary hospital, with 75 patients diagnosed with SIJ pain who underwent ultrasound-guided injection treatment between February 2021 and August 2024. Patients were stratified into different groups based on their clinical diagnoses and imaging findings. Pain intensity was assessed using the numeric rating scale (NRS) at baseline, 48 hours, one week, and three months postprocedure. A logistic regression model was applied to assess associations between predictive variables in univariate analysis and the likelihood of significant early pain reduction (≥70%). Variables were iteratively removed if p > 0.10, with the final model retaining predictors with p ≤ 0.05.​

Results:​ A cohort of 75 patients underwent 127 ultrasound-guided SIJ injections. Pain reduction of ≥50% was observed in 57.3% of patients at 48 hours postprocedure, declining to 34.7% at three months. SIJ pain and normal magnetic resonance imaging (MRI) ​significantly increased the likelihood of early pain reduction (OR = 5.13, 95% CI: 1.01-26.13, p = 0.049), and the presence of metabolic disorders (defined as the presence of diabetes mellitus and/or obesity) decreased the likelihood of early pain reduction (OR = 0.28, 95% CI: 0.09-0.95, p = 0.040). The median time to reintervention was 21 months. Metabolic disorders (HR = 2.72, p = 0.010) and cardiovascular comorbidities (hypertension, cardiac diseases, cerebrovascular diseases, peripheral arterial disease) (HR = 2.64, p = 0.011) were associated with a higher risk of earlier recurrence of pain, suggesting a role of systemic health in limiting treatment durability. The complication rate was low (2.67%), consisting only of transient sensory disturbances, with no major adverse effects identified.

Conclusions: Ultrasound-guided SIJ injections provided early pain reduction, with a complication rate of 2.67% limited to transient, self-limited sensory changes. Predictive factors, such as the presence of metabolic disorders, offer insights into patient selection and individualized treatment planning. A clinical diagnosis supported by a positive response to diagnostic injection may be sufficient to confirm SIJ-related pain, although further investigation is needed to elucidate the underlying cause.

## Introduction

Low back pain (LBP) is a widespread global health issue, with a point prevalence of 9.4% and an annual prevalence of 38% [1]. Sacroiliac joint (SIJ) pain is an often underdiagnosed cause of chronic LBP. It has been reported that approximately 25% of cases of lower back pain are related to the SIJ, stemming from various causes such as SIJ dysfunction, stress, pregnancy, trauma, lumbar fusion surgery, or bone grafting near the joint, and others [2].

SIJ pain arises from a dysfunction or inflammation of the SIJ and can be caused by mechanical issues (such as dysfunction caused by pregnancy); inflammatory conditions, namely, ankylosing spondylitis, psoriatic arthritis; and traumatic conditions (falls, car accidents, hip or lower back surgery) or degenerative conditions (osteoarthritis, degenerative disc diseases). Thus, its prevalence varies based on the underlying etiology [3,4]. 

While identifying the SIJ as a source of pain can be straightforward through clinical maneuvers and diagnostic injections, determining the underlying cause of SIJ pain remains challenging due to overlapping symptoms with other musculoskeletal or systemic conditions [5]. Reliable tests include the distraction, compression, thigh thrust, Gaenslen’s, and FABER (Patrick’s) tests. A positive diagnosis is established if at least three tests are positive, including either the thigh thrust or compression test, achieving 85% sensitivity and 76% specificity. The FABER test has the highest sensitivity (91.4%), while Gaenslen’s has the lowest (53%) [1,6,7]. 

Common imaging modalities include X-ray, CT, and MRI. X-ray, though widely used, has low sensitivity for early sacroiliitis, improving only after three to seven years of symptoms [8]. The New York criteria classify sacroiliitis from grade 0 (normal) to grade 4 (total ankylosis), indicating disease severity. CT is more sensitive than X-ray for chronic changes but cannot detect active inflammation and involves radiation exposure [9]. MRI is the gold standard for early inflammation detection and distinguishing active from chronic stages but is costly and not always accessible [10].

Acute sacroiliitis is characterized by subchondral bone marrow edema, joint effusion, and soft tissue inflammation [8]. SIJ pain management requires a multimodal approach, including pharmacological, integrative, and interventional treatments. Therapeutic options range from conservative measures to nerve blocks(such as lateral branch blocks of the SIJ and medial branch blocks of the dorsal rami of L4-S3), intra-articular injections, radiofrequency treatments, and surgery [11]. 

The American Society of Pain and Neuroscience (ASPN) recommends diagnostic intra-articular blocks to confirm SIJ pain, with a positive response defined as near-complete pain relief. A pain reduction of more than 70% on the numeric rating scale (NRS) following injection confirms the SIJ as the pain source [12]. 

Ultrasound-guided injection techniques are increasingly employed for their precision and safety, avoiding radiation exposure inherent in fluoroscopy-based methods [13]. Nevertheless, there are still significant gaps in our understanding of how patient-specific factors, including occupation, obstetrical history, and comorbidities, influence diagnosis and treatment outcomes [14]. 

This study aims to address gaps in the current understanding of SIJ pain management by integrating a stratification model based on clinical presentation and imaging findings. Given the diagnostic challenges and variable response to treatment, this study evaluates how patient characteristics influence outcomes of ultrasound-guided injection techniques. The study´s key objectives are (a) to establish a systematic stratification model for SIJ pain patients, (b) to assess the efficacy of ultrasound-guided injections, and (c) to identify predictors of significant pain relief, providing a more tailored approach to SIJ pain management. 

## Materials and methods

Study design and setting 

A retrospective observational study was conducted at the chronic pain unit (CPU) of a tertiary hospital in Portugal. The study design was structured to align with the three main objectives: patient stratification, treatment efficacy, and predictors of pain relief. To achieve this, we classified patients into diagnostic groups based on clinical findings and imaging results, analyzed the impact of ultrasound-guided injections on pain relief at multiple time points, and used predictive models to identify key factors influencing treatment success. The retrospective nature of the study allowed for a comprehensive analysis of real-world clinical outcomes, ensuring that findings are directly applicable to clinical practice. 

The study protocol was approved by the Department of Anesthesiology and the Ethics Committee on October 31, 2024 (nº 236-2024). Patient confidentiality was ensured in compliance with the General Data Protection Regulations (GDPR). The study adheres to the Strengthening the Reporting of Observational Studies in Epidemiology (STROBE) and Template for Intervention Description and Replication (TIDieR) guidelines for transparent reporting.  

Eligibility criteria 

​​The present study comprised a cohort of 78 patients diagnosed with SIJ pain who underwent ultrasound-guided injection techniques between February 2021 and August 2024. The inclusion criteria encompassed patients aged ≥18 years with a confirmed diagnosis of SIJ pain who underwent treatment with ultrasound-guided injection techniques.​ 

**Figure 1:**
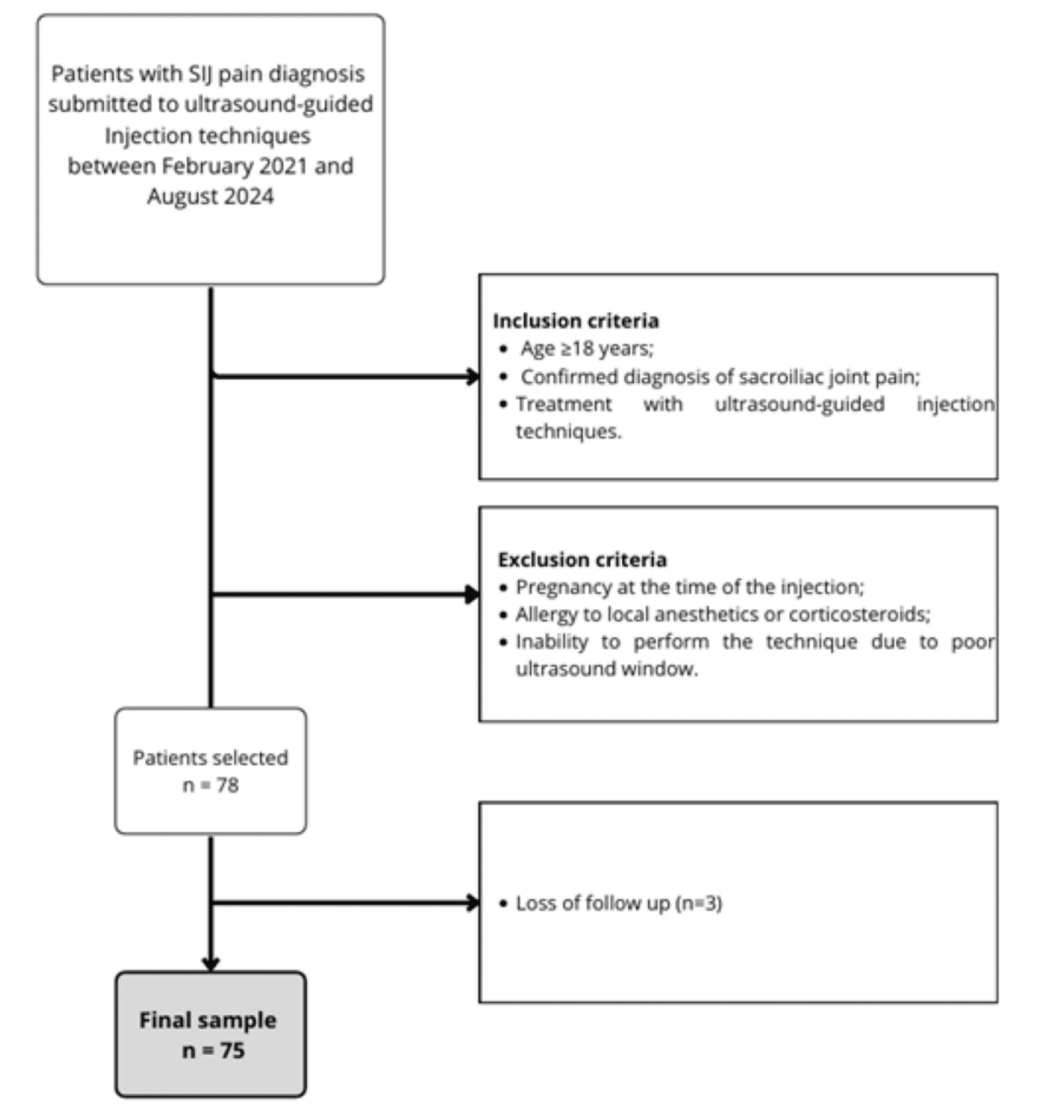
Patient sample selection flowchart

The exclusion criteria included current pregnancy at the time of the injection, allergy to local anesthetics or corticosteroids, and inability to perform the technique due to a poor ultrasound window. Following the exclusion of three patients due to loss of follow-up, the final sample consisted of 75 patients (Figure 1). 

Data collection 

Data were extracted from clinical records and structured within the following domains: (1) Sociodemographic factors included the patient´s age at first ultrasound-guided injection, gender, occupation, and educational level. Female-specific variables included the number of pregnancies, births, and delivery types. (2) Clinical variables included associated comorbidities such as rheumatologic disorders (rheumatoid arthritis, spondylarthritis, lupus, osteoarticular pathology, and fibromyalgia), metabolic pathology (defined as the presence of diabetes mellitus and/or obesity), cardiovascular disorders (hypertension, cardiac diseases, cerebrovascular diseases, peripheral arterial disease), psychiatric disorders (anxiety, depressive or eating disorders), previous low back surgical procedures, and pain intensity (NRS, 0-10). (3) Diagnostic variables included X-ray and MRI imaging of the SIJ. (4) Treatment variables included the use of physiotherapy, acupuncture, or other activities. (5) Procedural variables included a detailed record of the type of medication administered during the procedure (e.g., corticosteroids such as methylprednisolone, local anesthetics such as lidocaine or levobupivacaine), including dosage, combinations used, and the route of administration (intra-articular injection). Alternative therapies were separately categorized as physiotherapy or acupuncture. (6) Outcome variables included analgesic efficacy, defined as the percentage change in pain intensity measured using the NRS from baseline. Pain relief thresholds of ≥50% and ≥70% were evaluated at 48 hours, one week, and three months postprocedure. Additionally, the number of injections performed per patient was documented, along with the indications for postinjection radiofrequency (RF) and any procedure-related complications. Regarding these variables, missing data were observed only in the nonpharmacological treatment category (physiotherapy, acupuncture, hydrotherapy), with eight patients lacking this information in their clinical records. 

Patient stratification 

A testing protocol with three or more positive provocation tests was indicative of SIJ pain [6]. The Modified New York Scale was employed to stratify X-ray findings based on severity and disease progression (when available). In this stratification, a normal X-ray was defined as one without the previously mentioned changes suggestive of sacroiliitis, while an abnormal X-ray is characterized by the presence of such changes. The same method was applied to MRI, with a normal MRI showing no features consistent with sacroiliitis and an abnormal MRI exhibiting features consistent with sacroiliitis. Patients were stratified using a three-tiered approach based on clinical diagnosis, imaging findings (X-ray and MRI), and treatment response. The Modified New York Scale was used for X-ray evaluation, and MRI findings were classified as normal or abnormal based on the presence of edema in T2-weighted sequences. This stratification allowed for two parallel analyses: (a) a five-group descriptive classification based on diagnostic findings and (b) a two-group model distinguishing between normal and abnormal imaging findings. The goal was to determine whether imaging findings are associated with treatment efficacy and to assess whether a clinical diagnosis alone can guide effective treatment. 

A descriptive analysis was conducted in five groups: (A) clinical diagnosis only, (B) clinical diagnosis with normal imaging, (C) clinical diagnosis with MRI findings, (D) clinical diagnosis with X-ray findings, and (E) clinical diagnosis with both MRI and X-ray findings. A statistical analysis was conducted on two groups: (I) clinical diagnosis only with normal imaging findings and (II) clinical diagnosis with abnormal imaging findings (Table 1). 

**Table 1:** Diagnostic groups descriptive table

Diagnostic group	Total (n = 75)
Group I	25 (33.3%)
A	13 (17.3%)
B	12 (16.0%)
Group II	50 (66.6%)
C	8 (10.7%)
D	31 (41.3%)
E	11 (14.7%)

*Intervention Description* 

Intervention: This study used the ultrasound-guided injection techniques for the SIJ. 

Rationale: Ultrasound-guided injection techniques enable the targeted delivery of analgesics or corticosteroids to reduce inflammation and effectively manage pain. 

Consent: Prior to the procedure, the injection technique was thoroughly explained to the patient, and a written informed consent was obtained. 

**Figure 2:**
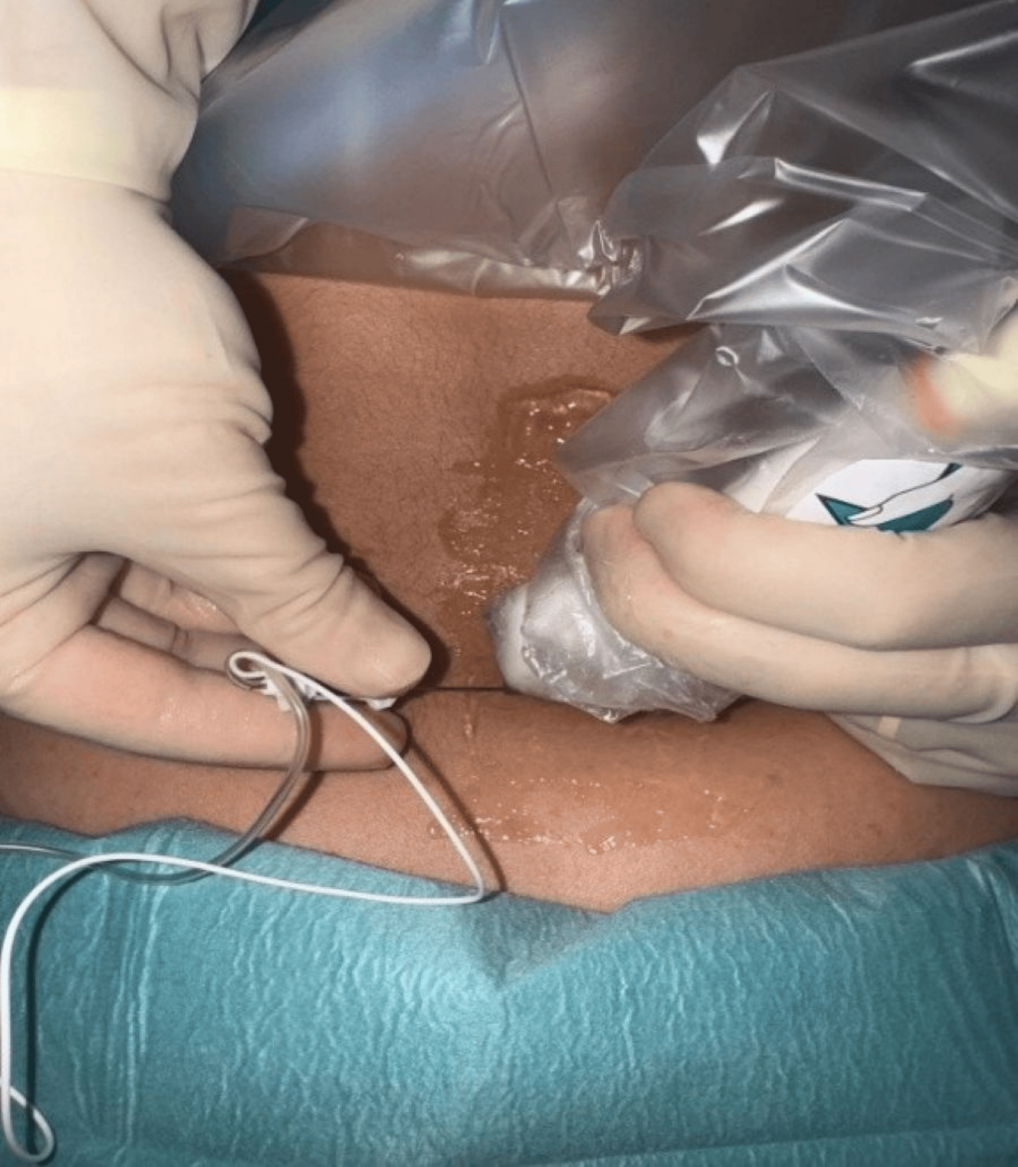
Ultrasound probe placement over the sacroiliac joint (SIJ) to achieve a short-axis view during the procedure

**Figure 3:**
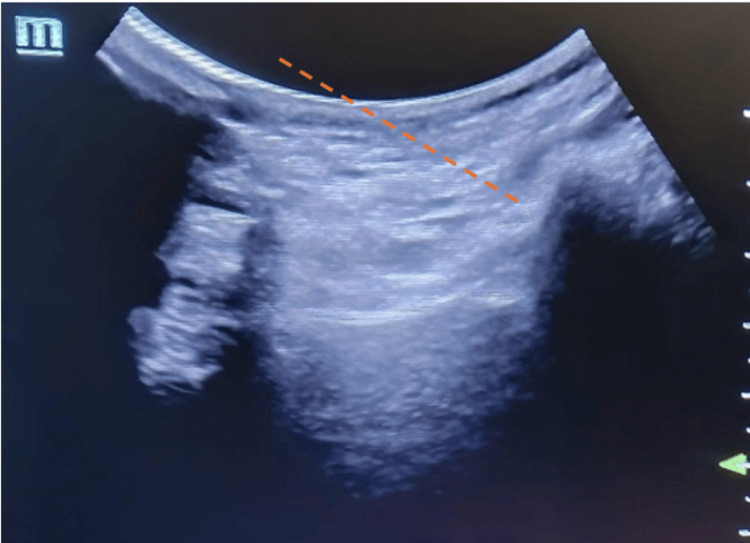
Ultrasound image of the sacroiliac joint The orange dashed line represents the path of the needle toward the sacroiliac joint

Procedural steps and materials: Patients were placed in a prone position, and the SIJ was identified using ultrasound, using a low-frequency curvilinear transducer, following antiseptic preparation with chlorhexidine 2% in an alcohol solution. A 22-gauge needle was inserted using an in-plane ultrasound-guided approach at approximately a 45º angle, targeting the anatomical projection of the SIJ space. A total of 3 mL of local anesthetic, either lidocaine 1% or levobupivacaine 0.25%, was administered, optionally combined with 1 mL (40 mg) of a corticosteroid (methylprednisolone or triamcinolone acetonide). Injections were performed using a 5 mL sterile syringe (Figures 2-3). The selection of an anesthetic and corticosteroid was based on availability and clinical discretion at the time of the procedure (Figures 2-3).

Who performed the technique: A senior anesthesiologist with expertise in ultrasound-guided pain interventions conducted the procedure.  

Patients were monitored for one hour to assess pain relief and side effects, such as local inflammation, transient pain exacerbation, or any allergic reactions.

Clinical outcomes 

Following ASPN recommendations, a significant early pain reduction (≥70% on the NRS at 48 hours postinjection) was considered the primary outcome and gold standard for confirming SIJ pain. 

Secondary outcomes included an early 50% decrease in NRS postprocedure; >50% pain response at one week and three months postprocedure; and time to re-intervention (relief of pain more than 50% in NRS), time until radiofrequency treatment, or indication for radiofrequency treatment.  

Although patients were monitored for one hour postprocedure for safety, this time point was not used for outcome analysis. Pain reduction was assessed at 48 hours to reflect the onset of corticosteroid action, in line with the study’s therapeutic rather than diagnostic focus.

Selection of the Representative Joint for Analysis 

For patients with bilateral injection, to simplify the analysis and focus on the patient as the primary unit, we selected the most clinically important SIJ for each patient based on the following criteria: 

Most painful joint: The joint with the highest NRS score was selected. 

Treatment response: If both sides required the same number of reinterventions, the joint with the lower reduction after the first injection was selected. If the reduction remained similar, the second injection was considered. 

Pain recurrence: The joint with the highest number of painful recurrences requiring reintervention was selected. 

This approach ensured that the analysis reflected the joint with the greatest clinical impact. In cases where both joints demonstrated identical outcomes, one was randomly selected to maintain consistency. 

By using this method, each patient contributed a single data point, representing their most painful joint, to the analysis. This approach avoids potential bias from including both joints, which could artificially inflate sample sizes and violate assumptions of independence in statistical analyses.  

Limitation to Four Injections 

For the purpose of this analysis, only the first four SIJ injections were considered per patient. This limitation was established to standardize the evaluation of treatment efficacy and durability of response across the study population. Patients who underwent more than four injections were included in the analysis, but data beyond the fourth injection were excluded to maintain consistency in comparisons. This approach ensures that the sample reflects a similar number of treatment events per patient, minimizing variability that could arise from differing numbers of interventions. 

Statistical analysis 

Statistical analyses were conducted using IBM SPSS Statistics for Windows, Version 29 (Released 2022; IBM Corp., Armonk, New York, United States) and R Statistical Software (v4.4.2; Released 2022) within RStudio. Descriptive statistics summarized demographic, clinical, and procedural data using means, medians, and percentages, with nonparametric tests applied due to nonnormal distributions. Univariate analyses examined the associations between predictors and outcomes using Chi-square or Fisher’s exact tests for categorical variables and Mann-Whitney U or Kruskal-Wallis tests for continuous variables. 

Longitudinal analyses assessed pain reduction across up to four SIJ injections using the Friedman test, with pairwise Wilcoxon signed-rank tests for comparisons at specific time points (Bonferroni correction, significance level 1.67%) [15]. Time to recurrence was evaluated using a Cox proportional hazards model with stepwise forward selection, incorporating right-censored cases for patients not receiving a second injection. Kaplan-Meier survival estimates were calculated, with group comparisons performed using the log-rank test, and proportional hazards assumptions verified through Schoenfeld residuals. 

A logistic regression model was used to assess the associations between sex, occupation, imaging findings, and the likelihood of significant early pain reduction (≥70%). Variables were iteratively removed if p > 0.10, with the final model retaining predictors with p ≤ 0.05. Model fit was evaluated using the Hosmer-Lemeshow test, ensuring at least 10 events per variable to minimize overfitting. Finally, the predictive value of different diagnostic stratifications (A-E, groups 1 vs 2, and MRI findings) was assessed by comparing the area under the ROC curves (AUCs) of the receiver operating characteristic (ROC) curves, with statistical differences evaluated using the DeLong test. 

## Results

Flow diagram 

We identified 78 potentially eligible patients, of which three were excluded due to loss of follow-up; the final sample included 75 patients, as shown in a detailed flowchart (Figure 1). 

Patient characteristics

The study included a total of 75 patients, of whom 58 (77.3%) were female. The median age at first infiltration was 56 years (50-66), with 10 (13.3%) needing bilateral synchronous infiltration. Among females, 52 (89.7%) had been pregnant, with nine having a previous history of miscarriage and eight of C-section birth. Regarding comorbidities, 38 (50.6%) had rheumatologic disorders, while 45 (60%) had a history of prior lower back surgery. Metabolic (38.7%) and cardiovascular diseases (25.3%) were also prevalent. Differences between diagnostic groups (Table 2) were nonsignificant for most sociodemographic variables, except for age (p = 0.038) and a higher proportion of tramadol use in Group 2 (p = 0.042) (Table 3). 

**Table 2:** Sociodemographic and patient characteristics-with univariate comparative analysis between diagnostic groups Note: Categorical variable values are presented as n (%). Continuous variables are presented using medians and interquartile ranges (percentile 25-percentile 75). Univariate analysis was performed using Chi-square (X^2^) or Fisher’s exact tests (*) for categorical variables and Mann-Whitney U (U) for continuous variables. A p-value of <0.05 indicates statistical significance

Patient characteristics	Total (n = 75)	Group 1 (n = 25)	Group 2 (n = 50)	Test statistics	p-value
Sociodemographic					
Age at 1st injection (years): p50 (p25-p75))	56	52	58.5	U = 809.5	0.038
Female (n = x (%))	58 (77.3%)	17 (68.0%)	41 (82.0%)	X^2 ^= 1.864	0.172
Educational qualifications					*0.855
Illiterate	1 (1.33%)	0 (0.0%)	1 (2.0%)		
1st-4th grade	30 (40.0%)	9 (36.0%)	21 (42.0%)		
5st-6th grade	16 (21.33%)	7 (28.0%)	9 (18.0%)		
7st-9th grade	15 (20.0%)	4 (16.0%)	11 (22.0%)		
10st-12th grade	9 (12.0%)	4 (16.0%)	5 (10.0%)		
Bachelor's degree	4 (5.33%)	1 (4.0%)	3 (6.0%)		
History of low back surgery	45 (60.0%)	14 (56.0%)	31 (62.0%)	X^2 ^= 0.250	0.617
Previous rheumatology consultation	24 (32.0%)	6 (24.0%)	18 (36.0%)	X^2 ^= 1.103	0.431
Comorbidities					
Rheumatological	38 (50.6%)	12 (48.0%)	26 (52.0%)	-	*0.467
Psychiatric	34 (43.33%)	8 (32.0%)	26 (52.0%)	X^2 ^= 2.690	0.141
Cardiovascular	19 (25.33%)	5 (20.0%)	14 (28.0%)	X^2 ^= 0.564	0.453
Metabolic	29 (38.67%)	9 (36.0%)	20 (40.0%)	X^2 ^= 0.112	0.737
Regular physical activity (any vs none)				-	*0.304
Gym	4 (5.33%)	1 (4.0%)	3 (6.0%)		
Cycling	2 (2.67%)	1 (4.0%)	1 (2.0%)		
Swimming	2 (2.67%)	1 (4.0%)	1 (2.0%)		
Yoga	1 (1.33%)	0 (0.0%)	1 (2.0%)		
Pilates	4 (5.33%)	0 (0.0%)	4 (8.0%)		

**Table 3:** Clinical characteristics of the participants, with univariate comparative analysis between diagnostic groups NSAIDs: nonsteroidal anti-inflammatory drugs Note: Categorical variable values are presented as n (%). Continuous variables are presented using medians and interquartile ranges (percentile 25-percentile 75). Univariate analysis was performed using Chi-square (X^2^) or Fisher’s exact tests (*) for categorical variables and Mann-Whitney U (U) for continuous variables. A p-value of <0.05 indicates statistical significance

Pain treatment (n = x (%))	Total (n = 75)		Group 1 (n = 25)		Group 2 (n = 50)	Test statistics	p-value
Pharmacological							
Paracetamol	30 (40.0%)		7 (28.0%)	23 (46.0%)		X^2 ^= 2.250	0.134
NSAIDs	39 (52.0%)		14 (56.0%)	25 (50.0%)		X^2 ^= 0.298	0.862
Tramadol	20 (26.66%)		3 (12.0%)	17 (34.0%)		X^2 ^= 4.125	0.042
Codeine	2 (2.66%)		1 (4.0%)	1 (2.0%)		-	*0.612
Tapentadol	38 (50.66%)		16 (64.0%)	22 (44.0%)		X^2 ^= 2.667	0.102
Oral morphine	8 (10.66%)		3 (12.0%)	5 (10.0%)		-	*0.999
Transdermal fentanyl	2 (2.66%)		1 (4.0%)	1 (2.0%)		-	*0.999
Transdermal buprenorphine	5 (6.66%)		2 (8.0%)	3 (6.0%)		-	*0.999
Duloxetine	9 (12.0%)		4 (16.0%)	5 (10.0%)		-	*0.470
Nonpharmacological							
Physiotherapy	40 (53.33%)		12 (48.0%)	28 (56.0%)		X^2 ^= 1.463	0.300
Acupuncture	14 (18.66%)		8 (32.0%)	6 (12.0%)		X^2 ^= 3.500	0.115
Hydrotherapy	25 (33.33%)		6 (24.0%)	19 (38.0%)		X^2 ^= 2.424	0.187
Injection characteristics (n = x (%))							
Time from diagnosis to injection	1 (0-9)						
Articulation injected						X^2 ^= 2.829	0.243
Left	35 (46.66%)						
Right	30 (40.00%)						
Both	10 (13.33%)						

Procedure characteristics

A total of 127 injections were performed: 63 on the right side and 64 on the left side. The median time from diagnosis to first injection was one (0-9) month. Regarding 1st injection: 32 (43%) were performed with lidocaine vs 43 (57%) with levobupivacaine, 55 (73%) plus methylprednisolone. 

Diagnosis stratification groups

Regarding sociodemographic and baseline clinical characteristics, diagnostic groups only differ in ongoing prescription of tramadol, with higher prescription frequency in patients with abnormal imaging findings (34.0% vs 12.0%, p = 0.042, Pearson Chi-Square).  

​​In univariate analysis, early significant pain relief (≥70%) showed no statistically significant association with any of the proposed diagnostic stratifications (5-group: p = 0.1633; 2-group: p = 0.112; MRI-based stratification: p = 0.0748; Fisher’s exact test). Considering early significant response as the gold standard for defining SIJ pain, we evaluated the predictive capacity of different diagnostic stratifications for SIJ pain. The AUC for the five-group stratification was compared to 0.85 (95% CI: 0.78-0.90) for clinical diagnosis combined with imaging findings. There were no differences between the AUCs (p = 0.220 for 5 vs 2, p = 0.464 for 5 vs MRI, p = 0.870 for 2 vs MRI, DeLong test).

Treatment efficacy

Following the first injection, a ≥50% pain reduction was achieved in 57.3% (n = 43) of patients. However, when using a ≥70% pain reduction threshold, the response rate dropped to 28% (n = 21). After one week, 54.7% (n = 41) maintained a ≥50% pain, and at three months, this percentage declined to 34.7% (n = 26). The median pain reduction (p25-75) at 48 hours, one week, and three months for the first injection was 57 ± (43-71), 56 ± (38-71), and 33 ± (13-63), respectively. 

Among 31 patients who proceeded to a second injection, pain relief patterns remained stable, with comparable responses observed at 48 hours and one week (Wilcoxon p = 0.744 and 0.703, respectively). The need for additional injection was not significantly associated with initial pain relief (p = 0.747 for ≥70% reduction, p = 0.099 for ≥50% reduction, Mann-Whitney U test), as summarized in Table 4 (pain score trajectory). 

**Table 4:** Pain score trajectory Note: Relative frequencies refer to the total sample (75 patients) for each injection and the total number of patients who underwent that sequence of injections. Values are presented as n (%) or median (P25-P75). P-values are from pairwise comparisons using Wilcoxon signed-rank tests (W) with Bonferroni correction (*p < 0.0167), following the application of Friedman’s test (F). p-value*: this p-value uses 48 hours as reference (ref); p-value**: this p-value uses one week as reference-not applicable. a) Only two patients proceeded to the 4th injection-the small sample size limits statistical interpretation

Numeric rating scale (NRS)	N (%)	Median (P25-P75)	Test statistics	p-value*	p-value**
Pain reduction-1st Injection	75 (100)		W = 50.844	p < 0.001	
48 hours		57 ± (43-71)		ref	-
1 week		56 ± (38-71)	F = 0.327	0.744	ref
3 months		33 ± (13-63)	F* = 5.185|	<0.001	<0.001
			F** = 4.858		
Pain reduction-2nd Injection	31 (41.3)		W = 11.455	p = 0.003	
48 hours		57 ± (44-78)		ref	-
1 week		57 ± (44-75)	F = -0.381	0.703	ref
3 months		50 ± (14-75)	F* = 2.096|	0.036	0.013
			F** = 2.477		
Pain reduction-3rd Injection	5 (6.67)		W = 2.714	p = 0.257	
48 hours		63 ± (38-71)		ref	-
1 week		63 ± (53-69)	F = 0.158	0.874	ref
3 months		38 ± (0-63)	F* = 1.265|	0.206	0.268
			F** = 1.107		
Pain reduction-4th Injection	2 (2.67)		-		
48 hours		a)			
1 week					
3 months					

Patients who underwent a second injection did so after a median of 11 months (IQR: 4-19 months). Those proceeding to a third or fourth injection required further treatment within 6-8 months. Across all injections, short-term relief at 48 hours remained consistent (p = 0.392, Friedman test). However, long-term responses at three months showed a decline after the third injection onward (p = 0.036 for the second injection, p = 0.206 for the third, Wilcoxon test). A total of 22 patients were indicated for radiofrequency (RF) treatment, with 19.3% (n = 6) ultimately undergoing thermal RF (Table 5). Complications remained rare across all injections (2.67% (n = 2)). 

**Table 5:** Treatment efficacy (TE) RF: radiofrequency Note: Descriptive data only. Relative frequencies refer to the total sample (75 patients) for each injection and the total number of patients who underwent that sequence of injections. Values are presented as n (%)

Injection	1st	2nd	3rd	4th
Number (n = x (%))	75 (100)	31 (41.3)	5 (6.7)	3 (4.0)
Treatment characteristics				
Lidocaine	32 (42.7)	24 (77.4)	2 (40.0)	2 (66.7)
Levobupivacaine	43 (57.3)	7 (22.6)	3 (60.0)	1 (33.3)
Methylprednisolone	55 (73.3)	28 (90.3)	4 (80.0)	3 (100.0)
Treatment efficacy				
Early ≥50%	43 (57.3)	19 (61.3)	4 (80.0)	2 (100.0)
Early ≥70%	21 (28.0)	11 (35.5)	1 (20.0)	0 (0.0)
1 week ≥50%	41 (54.7)	19 (61.3)	4 (80.0)	2 (100.0)
3 month ≥50%	26 (34.7)	13 (41.9)	2 (40.0)	2 (100.0)
Indications for RF	8 (10.7)	10 (32.3)	2 (40.0)	2 (66.7)
Evolution to RF	0 (0.0)	6 (19.3)	0 (0.0)	0 (0.0)
Complications	1 (1.3)	2 (6.5)	0 (0.0)	0 (0.0)

Predictors of early ≥ 70% pain reduction 

Multivariable logistic regression identified predictors of a significant response to the first injection (≥70% reduction in NRS). Only variables with p ≤ 0.2 in univariate analysis were included. The final model presented below corresponds to the MRI-based stratification, excluding patients without MRI data. Using the backward conditional selection method (entry p = 0.1, removal p = 0.05), the model demonstrated acceptable performance, with the following predictors: 

Patients with normal or absent MRI findings were 5.13 times more likely to achieve a significant response compared to those with abnormal MRI findings (OR = 5.13, 95% CI 1.01-26.13, p = 0.049). Patients with metabolic diseases (e.g., corticosteroid use) were less likely to achieve a significant response (OR = 0.28, 95% CI 0.09-0.95, p = 0.040). Conversely, the enter method demonstrated a poor model fit (Hosmer-Lemeshow: p = 0.003) and is not presented in detail.

Predictors of time to reintervention/first recurrence

As reported in the methods, a Kaplan-Meier method was used to evaluate time to recurrence, as a survival analysis. The mean time to recurrence was 23 months (95% CI: 19-27) with a standard error of 2.05. The median time to recurrence was 21.00 months (95% CI: 16-25), with a standard error of 2.15. The mean time to recurrence is limited to the largest censored observation, as per standard practice. These results indicate that 50% of patients experienced a recurrence within 21 months after the first injection, with 25% recurring within 13 months and 25% surviving at least 38 months without a second injection. 

Cox proportional hazards regression, with forward stepwise selection, identified two significant predictors of time to second injection (χ² = 26.637, p <0.001, Wald test 10.96, p = 0.004). Metabolic disorders (HR = 2.72, 95% CI: 1.28-5.81, p = 0.010) and cardiovascular pathologies (HR = 2.64, 95% CI: 1.25-5.57, p = 0.011) were associated with a higher hazard of reintervention. The Schoenfeld residuals test showed no evidence of proportional hazards violation for any other covariates (global p = 0.123). However, the global test did indicate a significant violation in the overall model (p = 0.028). Residual plots and potential interactions with time were explored to address this inconsistency. Given the observed violation, an Aalen additive model was applied to assess time-varying effects on the covariates. The findings from the Aalen additive model reveal clinically relevant dynamics in risk prediction. The Aalen additive model revealed that the cumulative effects of metabolic disorders approached significance (p = 0.087), while cardiovascular pathologies were not significant (p = 0.141). 

Complications

Complications associated with the procedure were also evaluated in this study. No true complications were observed following the 127 ultrasound-guided SIJ injections. Three patients (2.4%) reported transient sensory effects in the posterior pelvic region (mild numbness or tingling), which resolved spontaneously within 24 to 72 hours without intervention. These effects were consistent with the expected pharmacologic action of local anesthetics and have therefore not been classified as complications.

## Discussion

LBP is a major global health concern [1]. SIJ pain is a common yet frequently underdiagnosed cause of chronic low back pain. Accurate diagnosis remains challenging due to symptom overlap with other musculoskeletal conditions, with the need to use both imaging studies and clinical maneuvers [2,5]. This study evaluates patient stratification based on clinical and imaging data to assess the efficacy of ultrasound-guided SIJ injection, establish an optimal treatment plan, and identify pain predictors. 

Stratification based on clinical and imaging findings has provided valuable insights into diagnostic and prognostic factors that impact treatment outcomes. The use of standardized methodologies in accordance with STROBE and TIDieR guidelines ensures reproducibility and a robust interpretation of data. 

Findings

In our study, patients with abnormal imaging findings demonstrated a significantly higher prescription rate of tramadol, with no corresponding increase in anti-inflammatory drug use. This pattern suggests that additional pain sources, distinct from the SIJ, may contribute to their symptoms. Furthermore, it may indicate a potential selection bias, where individuals with more severe or refractory pain were preferentially selected for intervention. This potential bias should be taken into consideration when interpreting the clinical outcomes. Future research is warranted to determine whether this subgroup represents a clinically distinct phenotype of SIJ-related pain.

The study demonstrated an early pain reduction of ≥50% in 57.3% of patients at 48 hours, consistent with previous research on SIJ interventions​ [16]. The 48-hour timepoint was deliberately chosen to assess the therapeutic effect of the corticosteroid, rather than the transient anesthetic response typically observed within the first hour postprocedure. Although 34.7% of patients maintained a ≥50% pain reduction at three months, we acknowledge that this improvement is unlikely to be directly attributable to the pharmacologic effect of the injection, as both local anesthetics and corticosteroids have relatively short durations of action. Instead, sustained pain relief at this time point may reflect partial or complete resolution of the underlying SIJ dysfunction, modulation of inflammatory processes, placebo effects, improved function, or the cumulative benefit of adjunct therapies such as physiotherapy.

This finding is consistent with the understanding that SIJ pain, like other chronic musculoskeletal conditions, has a multifactorial and fluctuating course, and that interventional procedures may facilitate recovery by interrupting the pain cycle or improving function, even if the pharmacological effect is transient. We have therefore interpreted the three-month outcomes as functional and patient-centered endpoints, rather than as a direct measure of drug efficacy [17]. 

Interestingly, patients with normal or absent MRI findings were more likely to achieve early pain reduction. This association may partly reflect selection bias, as a large proportion of patients did not undergo MRI. Nonetheless, even in a univariate analysis restricted to the 29 patients who underwent MRI, an abnormal MRI was significantly associated with a lower likelihood of response.

This finding challenges the conventional reliance on positive imaging results for treatment decisions [18] and suggests that clinical presentation may be a more reliable indicator for patient selection in SIJ interventions. This is consistent with the most recent studies on the diagnosis of SIJ pain that state that a positive block of SIJ is mandatory for a definite diagnosis [11,19]. 

Beyond imaging findings, metabolic disorders emerged as a significant factor influencing treatment outcomes. The presence of metabolic disorders was found to decrease the likelihood of pain reduction and increase the risk of earlier recurrence, emphasizing the importance of addressing comorbidities in pain management. This supports the growing evidence linking metabolic disorders to chronic pain and treatment resistance, suggesting a personalized, holistic approach to pain management that includes both joint pathology and systemic health [20]. Furthermore, metabolic and cardiovascular pathologies were identified as predictors of earlier reintervention, highlighting the complexity of chronic pain as a multisystemic condition. These comorbidities may affect pain perception, tissue healing, and treatment response through mechanisms such as chronic inflammation and altered pain processing [20]. Intriguingly, patients with rheumatological diseases, either immune-mediated or not, did not have a different response to SIJ injection. This may be explained by the fact that our population had varying degrees of disease severity, different medications, with some patients being treated with biological disease-modifying drugs, and different imaging evaluations. All these factors may have limited our analysis of this subgroup. 

Patients with a history of low back surgery and SIJ pain exhibited no improvement in their pain complaints following ultrasound-guided SIJ injection. It is well-known that lumbar pain is often multifactorial, and this may be a potential explanation for this finding [21]. 

The mean time to reintervention was 21 months, indicating a prolonged duration of pain relief for many patients. This finding provides important insights for patient counselling and follow-up planning. Notably, this duration is compared to or longer than previously reported outcomes [22]. This difference may be explained by variations in patient demographics or treatment approaches. However, this finding may be influenced by factors such as patient nonattendance, postponement of appointments, or other delays in treatment.

The absence of procedural complications in our cohort supports the safety of ultrasound-guided SIJ injections. Although three patients experienced temporary changes in sensitivity near the injection site, these were mild, self-limited, and consistent with the anticipated effect of local anesthetic agents, rather than true complications. This distinction is important when evaluating the risk profile of the procedure and highlights the low incidence of adverse events in this population [23]. 

Our study's stratification approach provides a nuanced framework for patient assessment when considering both clinical and imaging findings. These findings have important implications for clinical practice.

Patient Selection

While SIJ injections appear to be effective for many patients, those with metabolic disorders may require a multidisciplinary management approach [20]. 

Imaging Utilization

The predictive value of normal MRI findings suggests that clinical assessment should be prioritized over imaging results when considering SIJ interventions [16]. 

Long-Term Management

Given the gradual decline in efficacy over time, clinicians should develop comprehensive long-term management plans, potentially incorporating lifestyle modifications and targeted therapies to address metabolic and cardiovascular health [22]. 

Follow-Up Strategies

The variability in time to reintervention highlights the need for individualized follow-up protocols, with closer monitoring for patients with identified risk factors for earlier recurrence [20]. 

Limitations and future directions

Although our study benefits from a relatively large sample size and comprehensive analysis, it is limited because the single-center design may reduce the generalizability of results to other clinical settings. As a retrospective study, it relies on historical clinical records, which may be incomplete or subject to recall bias.

The study did not include a comparative analysis with other treatment modalities, such as RF or conservative management strategies. The primary aim was to evaluate the efficacy of ultrasound-guided SIJ injections in a real-world clinical setting. However, the absence of a direct comparison with alternative interventions limits the ability to determine the relative effectiveness of ultrasound guidance versus other approaches.

In addition, the efficacy of the treatment was evaluated solely through pain intensity, without incorporating standardized functional outcome measures. This was due to the retrospective nature of the study and the lack of routinely collected functional assessments in clinical records. Although pain relief is a clinically relevant and patient-centered outcome, the lack of functional data limits a broader understanding of the intervention's impact on daily activities and quality of life.

Another relevant limitation is the heterogeneity in the anesthetic agents used across procedures. While both lidocaine and levobupivacaine were administered in similar volumes, their pharmacokinetic properties differ. However, given that pain outcomes were assessed 48 hours postprocedure, beyond the expected duration of action of either anesthetic, it is unlikely that this variability significantly influenced the primary outcome. Still, this heterogeneity reflects real-world clinical practice and was not intended to serve as a comparative analysis between agents.

Furthermore, the variability in time to reintervention observed among patients emphasizes the need for individualized follow-up protocols, especially for those with identifiable risk factors for earlier recurrence. A standardized follow-up timeline may not adequately capture the clinical course in all patient profiles.

Further research should focus on (1) conducting comparative studies or randomized clinical trials to evaluate the relative efficacy of different therapeutic approaches for SIJ pain; (2) exploring the cost-effectiveness of imaging modalities in guiding treatment and conducting long-term follow-up to evaluate sustained efficacy and recurrence rates; (3) investigating the mechanisms-such as inflammatory processes-through which metabolic, rheumatologic and cardiovascular comorbidities impact pain chronicity and treatment response; (4) developing and evaluating targeted interventions aimed at prolonging pain relief, particularly in high-risk patient populations [24]; (5) investigating the potential role of biomarkers or advanced imaging techniques in enhancing patient selection and predicting treatment outcomes [16]; and (6) exploring the clinical characteristics, pain mechanisms, and treatment responses of patients with higher baseline opioid (tramadol) prescription rates, to better understand potential selection biases and optimize therapeutic strategies.

## Conclusions

This study suggests that early interventional treatment guided by ultrasound has a positive impact on patients with SIJ pain, particularly those with normal MRI findings. Patients with metabolic disorders (defined as the presence of diabetes mellitus and/or obesity) may benefit from multimodal strategies that include optimized management of these comorbidities alongside SIJ injections, as such approaches may reduce the risk of early pain recurrence. The ultrasound-guided injection techniques used in this study appear to be a safe and effective second-line treatment option for SIJ pain, with a low complication rate consisting solely of transient sensory disturbances and no major adverse events. Finally, our findings support the use of diagnostic SIJ injections as a valid tool to confirm the clinical diagnosis of SIJ-related pain, potentially enabling earlier therapeutic intervention without the need to rely exclusively on imaging. This approach may help optimize treatment timelines, improve patient outcomes, and enhance satisfaction in routine clinical practice.
